# Preparation of a Titania/X-Zeolite/Porous Glass Composite Photocatalyst Using Hydrothermal and Drop Coating Processes

**DOI:** 10.3390/molecules20022349

**Published:** 2015-01-30

**Authors:** Atsuo Yasumori, Sayaka Yanagida, Jun Sawada

**Affiliations:** 1 Department of Materials Science and Technology, Tokyo University of Science, 6-3-1 Niijuku, Katsushika-ku, Tokyo 125-8585, Japan; E-Mails: YANAGIDA.Sayaka@nims.go.jp (S.Y.); bb_is_my_life_tr@yahoo.co.jp (J.S.); 2 Photocatalysis International Research Center, Research Institute for Science and Technology, Tokyo University of Science, 2641 Yamazaki, Noda-shi, Chiba 278-8510, Japan

**Keywords:** TiO_2_, hydrothermal, X-zeolite, Porous-glass, melt-quenching, composite, photocatalyst, adsorption, 2-propanol

## Abstract

Combinations of TiO_2_ photocatalysts and various adsorbents have been widely studied for the adsorption and photocatalytic decomposition of gaseous pollutants such as volatile organic compounds (VOCs). Herein, a TiO_2_-zeolite-porous glass composite was prepared using melt-quenching and partial sintering, hydrothermal treatment, and drop coating for preparation of the porous glass support and X-zeolite and their combination with TiO_2_, respectively. The obtained composite comprised anatase phase TiO_2_, X-zeolite, and the porous glass support, which were combined at the micro to nanometer scales. The composite had a relatively high specific surface area of approximately 25 m^2^/g and exhibited a good adsorption capacity for 2-propanol. These data indicated that utilization of this particular phase-separated glass as the support was appropriate for the formation of the bulk photocatalyst-adsorbent composite. Importantly, the photocatalytic decomposition of adsorbed 2-propanol proceeded under UV light irradiation. The 2-propanol was oxidized to acetone and then trapped by the X-zeolite rather than being released to the atmosphere. Consequently, it was demonstrated that the micrometer-scaled combination of TiO_2_ and zeolite in the bulk form is very useful for achieving both the removal of gaseous organic pollutants and decreasing the emission of harmful intermediates.

## 1. Introduction

Various atmospheric pollutants in the environment continue to cause considerable problems. In particular, volatile organic compounds (VOCs) such as formaldehyde, acetaldehyde, acetone, 2-propanol, and toluene, released mainly from paints and adhesives used in building and construction materials, give rise to sick building syndrome. Titanium dioxide (TiO_2_) photocatalysts are well known to decompose organic compounds under ultraviolet (UV) light irradiation via oxidation reactions, and therefore, have been widely studied and are practically employed in combination with “black lights”, such as fluorescent lamps, UV lamps, and sunlight to remove indoor VOCs [1,2,3,4,5,6,7,8,9].

The concentration of VOCs in living spaces is typically very low at several tens of ppm (by volume and used hereafter) to less than 1 ppm [10]. Under such conditions, a TiO_2_ photocatalyst in the form of a coating material has two main disadvantages. First, the decomposition rate is not sufficiently high for gaseous pollutants at very low concentrations in the atmosphere, because the photocatalytic reaction mediated by TiO_2_ (or any other solid semiconductor photocatalyst) occurs at or very close to the surface, and the diffusion process for the gaseous pollutants from the atmosphere to the surface of the TiO_2_ is rate limiting. Second, the removal of VOCs is not effective under weak intensity radiation or during the nighttime. 

One potential solution for overcoming such disadvantages of TiO_2_ is the combination of a TiO_2_ photocatalyst with an appropriate adsorbent. In such a composite, the pollutants are first concentrated in the pores of the adsorbent, and then photocatalytic degradation of the adsorbed or desorbed pollutants proceeds on the TiO_2_ or in the pores due to the generation of oxygen radicals following light irradiation. Because the advantages of combining TiO_2_ with an adsorbent are also observed for the degradation of dilute concentrations of pollutants in waste water, various adsorbents have been combined with TiO_2_ for the rapid removal of low levels of pollutants in the environment [11]. For example, studies have been reported on the combination of TiO_2_ photocatalysts with ceramic supports [12,13,14,15,16], porous glasses and glass fiber [17,18,19,20,21], activated carbon [22,23,24,25,26] and graphene [27,28,29,30], silica gel [31,32,33,34,35] and other mesoporous materials [36,37,38,39,40]. 

Zeolites are another important category of effective adsorbents that have been combined with TiO_2_. Natural zeolite is an aluminosilicate mineral with micro pores. Various synthetic zeolites have been prepared with controllable pore sizes and surface properties and used as molecular sieves for the adsorption of molecules of particular sizes and polarities [41]. In addition, there are many studies of combinations of TiO_2_ photocatalysts and zeolites for photochemical and photocatalytic science and applications [42,43]. For, example, studies have been reported on the adsorption and photocatalytic degradation of various organic pollutants [44,45,46,47,48,49,50] and nitrogen oxides (NO_x_) [51,52]. Among the synthesized zeolites, X- and Y-zeolites, which have faujasite (FAU) structure, are well known to have large pores (0.74 nm in diameter) due to their super cage structures and the ability to adsorb relatively large organic molecules [53]. Notably, the adsorption properties of those FAU-type zeolites are influenced by their compositions, and particularly the Si/Al ratio. The number of Na^+^ (or other alkaline or alkaline earth metal) ions accompanying the [AlO_4_]^−^ tetrahedra on the surface of X-zeolite is relatively high because of its low Si/Al ratio (Si/Al ≈ 1.25) compared to that of other FAU-type zeolites, such as Y-zeolite (Si/Al ≈ 2.3) [53]. Because cations on the surfaces of zeolite pores exhibit strong electrostatic interactions with molecules having dipole or quadrupole moments, X-zeolite can adsorb molecules, such as H_2_O and lower alcohols. Therefore, in the present study, X-zeolite with its high ability for the adsorption of polar VOCs was selected as the adsorbent for the model polar VOC pollutant 2-propanol.

For preparation of a composite comprising TiO_2_ and X-zeolite, three important points were considered. (1) It is necessary for the X-zeolite to be adjacent to the TiO_2_ to ensure that photo-generated oxygen radicals diffuse and attack the VOCs adsorbed in the X-zeolite before they are deactivated [54,55]; (2) TiO_2_ and the X-zeolite should be co-loaded on appropriate supports to enable easy handling in practical applications [56]; (3) A transparent support for the TiO_2_ and X-zeolite is required to allow passage of near-UV light for photoexcitation of the TiO_2_. Previous studies mentioned above could achieve the first point that the zeolite was adjacent to the TiO_2_ by use of mainly commercial particulate zeolite and appropriate synthesis procedures of TiO_2_ fine particles. However, for the practical application, the above points (2) and (3) are essential. In addition, the support should also be porous to provide a highly dispersive condition of zeolite which can result in a large adsorption and reaction area. Therefore, porous silicate glass was selected because of its high specific surface area, transparency in the visible and UV range, and relatively high chemical durability, and zeolite was directly synthesized on the glass support. The disk-shaped silicate glass supports were prepared via the partial sintering of silicate glass frits comprising Na_2_O-B_2_O_3_-CaO-ZrO_2_-Al_2_O_3_-SiO_2_ with ratios in the immiscibility region to achieve phase separation [57]. The porous glass was formed during subsequent hydrothermal treatment, which was used for the synthesis of the X-zeolite on the glass support. This process was expected to make the zeolite combine with the porous support much rigidly. The TiO_2_ was then coated on the X-zeolite on the glass support by dropping a titanium alkoxide solution into the zeolite-porous glass composites. The crystalline phases and microstructures of the obtained TiO_2_-coated zeolite-porous glass composites were characterized and their ability to adsorb and photocatalytically decompose 2-propanol was examined.

## 2. Results and Discussion

### 2.1. Crystalline Phases of the Composites

**Figure 1:**
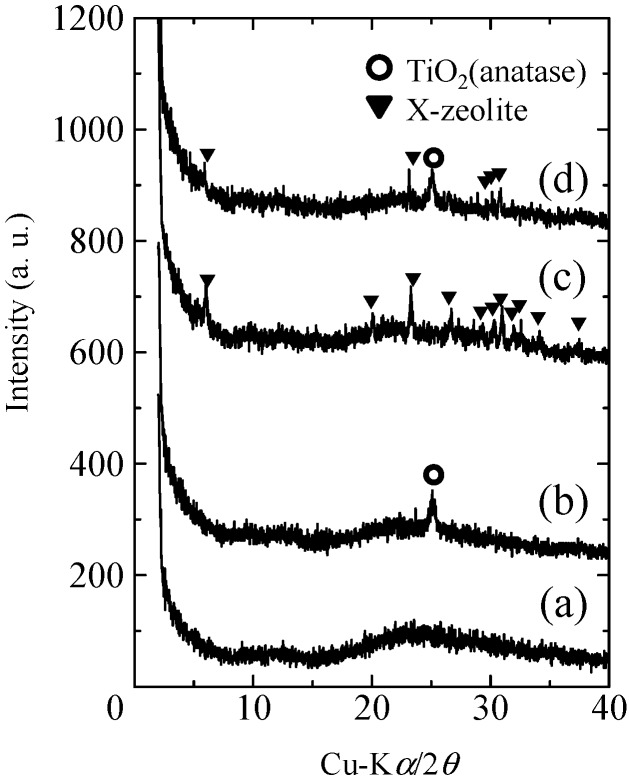
XRD patterns of the prepared samples: (a) partially sintered glass; (b) TiO_2_-coated partially sintered glass; (c) zeolite-porous glass composite; and (d) TiO_2_-coated zeolite-porous glass composite.

The XRD patterns of the partially sintered glass and composite samples are shown in Figure 1. The partially sintered glass exhibited only a halo pattern in the diffraction pattern (Figure 1a,b), indicating that the glass was not crystallized during the sintering process. The diffraction pattern of the zeolite-porous glass composite (Figure 1c) contained diffraction peaks for the X-zeolite of a FAU structure, while that for the TiO_2_-coated composite sample (Figure 1d) exhibited the diffraction peak for anatase TiO_2_ in addition to those for the X-zeolite, although the intensities of the latter peaks were slightly decreased. These results indicated that the X-zeolite precipitated following hydrothermal treatment and remained in the composite following the TiO_2_ coating process.

### 2.2. Micro Textures of the Composites

**Figure 2:**
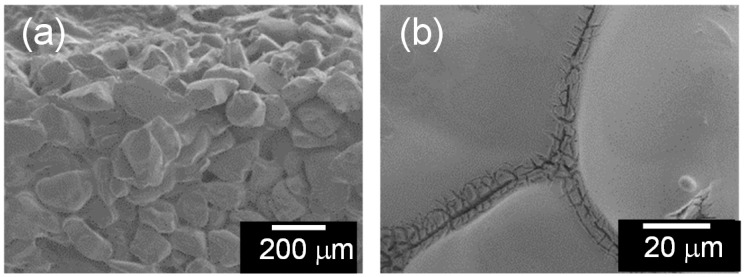
FE-SEM images of the TiO_2_-coated partially sintered glass with (**a**) low and (**b**) high magnification.

**Figure 3:**
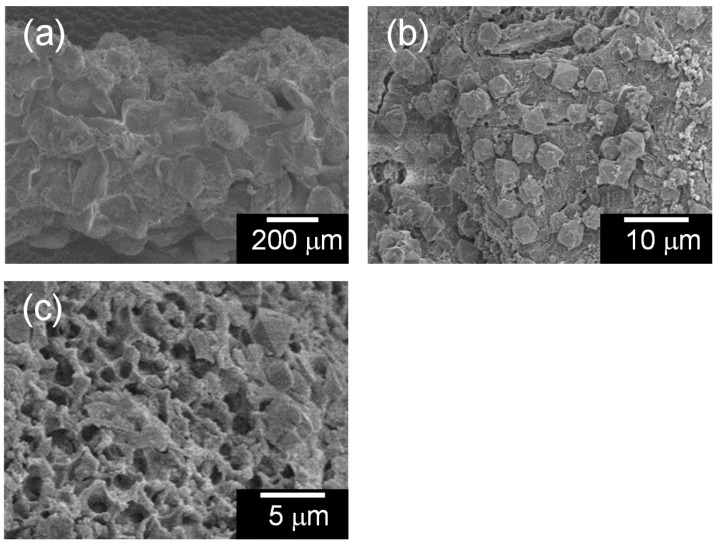
FE-SEM images of the zeolite-porous glass composite: (**a**) low magnification image; (**b**) precipitated zeolite particles; and (**c**) micro pores formed on the glass surface.

**Figure 4:**
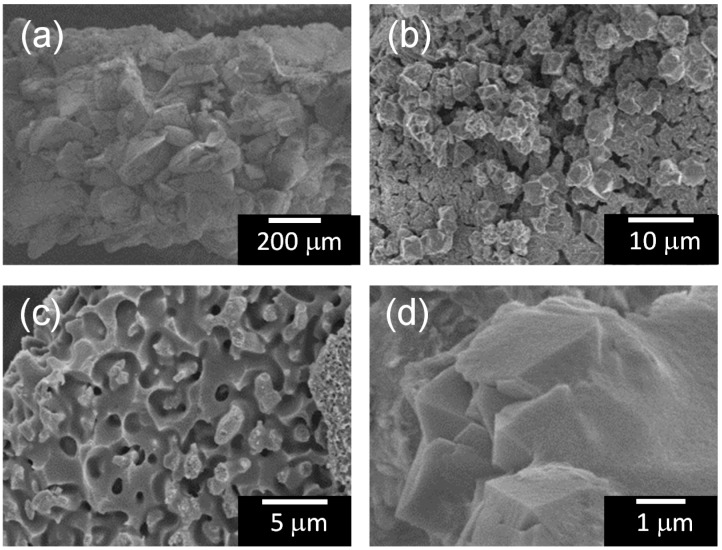
FE-SEM images of the TiO_2_-coated zeolite-porous glass composite: (**a**) low magnification image; (**b**) precipitated zeolite particles; (**c**) micro pores formed on the glass surface; and (**d**) TiO_2_ coating layer on the zeolite.

The micro textures of the TiO_2_-coated partially sintered glass, the zeolite-porous glass composite, and the TiO_2_-coated composite were observed via FE-SEM, and the results are shown in Figure 2, Figure 3 and Figure 4, respectively. The TiO_2_-coated partially sintered glass disk comprised glass particles with diameters of 100–200 μm (Figure 2a), which were similar to those of the raw glass frit particles. Sufficient spaces remained through which 2-propanol gas could diffuse to the interior of the disk. In addition, many small cracks were observed at the interfaces between the glass particles due to precipitation of the thick TiO_2_ layer (Figure 2b). Interestingly, the zeolite-porous glass composite had a similar texture to that of the partially sintered glass on a macroscopic level, as can be seen in Figure 3a. However, in the microstructure near the surface that was directly exposed to the solution during hydrothermal treatment, many cubic precipitates were formed (Figure 3b), which were assigned to the X-zeolite based on their cubic shape [58] and the results of the XRD analysis. Micro pores formed by the selective etching of the phase separation texture during the hydrothermal treatment were also detected on the glass grains, as can be seen in Figure 3c. Finally, a thick TiO_2_ layer covered the surface of the micro structure in the coated composite, but the texture of the sample was not changed significantly, as can be seen in Figure 4a–c. Figure 4d shows the coated X-zeolite as an example. 

### 2.3. Adsorption Characteristics of the Composites

**Figure 5:**
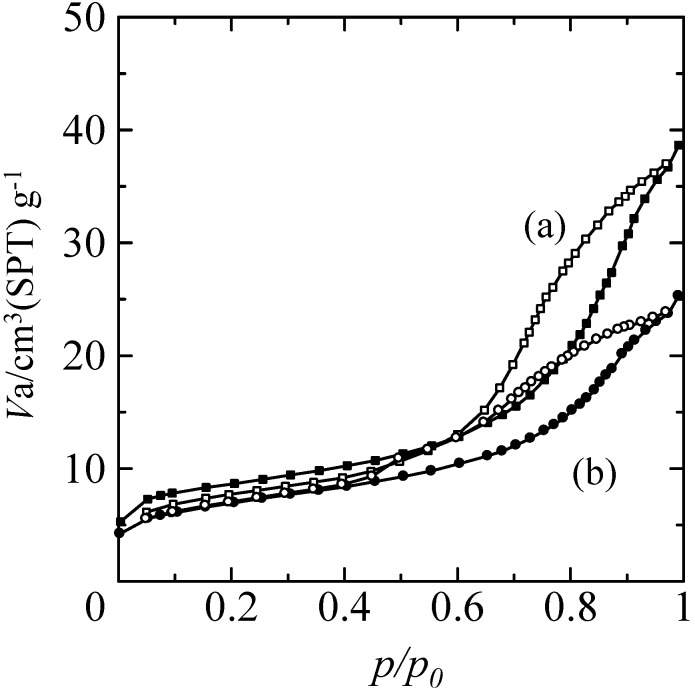
Adsorption and desorption isotherms of the zeolite-porous glass composite- (a) before and (b) after TiO_2_ coating.

The adsorption and desorption isotherms of the zeolite-porous glass composite before and after TiO_2_ coating are shown in Figure 5. Both samples exhibited definite hysteresis in the high relative pressure region, and these isotherms corresponded to type IV isotherms as classified by International Union of Pure and Applied Chemistry (IUPAC) [59]. Typical FAU type zeolites exhibit type II isotherms with little or no adsorption and hysteresis in the same region [59,60]. In contrast, type IV isotherms are typically observed in silica gel or mesoporous silica [59]. Therefore, these isotherms indicated that the adsorption behavior of the composite could be attributed to the micropores of the zeolite and the mesopores of the porous glass in the low and the high relative pressure regions, respectively. Not surprisingly, the adsorption volume of the TiO_2_ coated sample was significantly decreased compared to that of the non-coated composite in the high relative pressure region. The multipoint Brunauer–Emmett–Teller (BET) specific surface area (Sg) of the zeolite-porous glass composite was approximately 30 m^2^/g or approximately 4% that of a commercial X-zeolite (approximately 780 m^2^/g, F9, Tosoh Co., Tokyo, Japan). After TiO_2_ coating, the samples retained a high Sg of approximately 25 m^2^/g. These results indicated that the deposited TiO_2_ prevented the N_2_ gas from being adsorbed in the mesopores of the porous glass but allowed the gas to be adsorbed in the micropores of X-zeolite, and thus the combination of a TiO_2_ photocatalyst and X-zeolite adsorbent in a composite was successfully achieved. The Sg of the TiO_2_-coated partially sintered glass was less than the detection limit of the measurement system, suggesting that the coated TiO_2_ layer did not contribute to the adsorption of 2-propanol or acetone as an intermediate oxidative product of 2-propanol.

**Figure 6:**
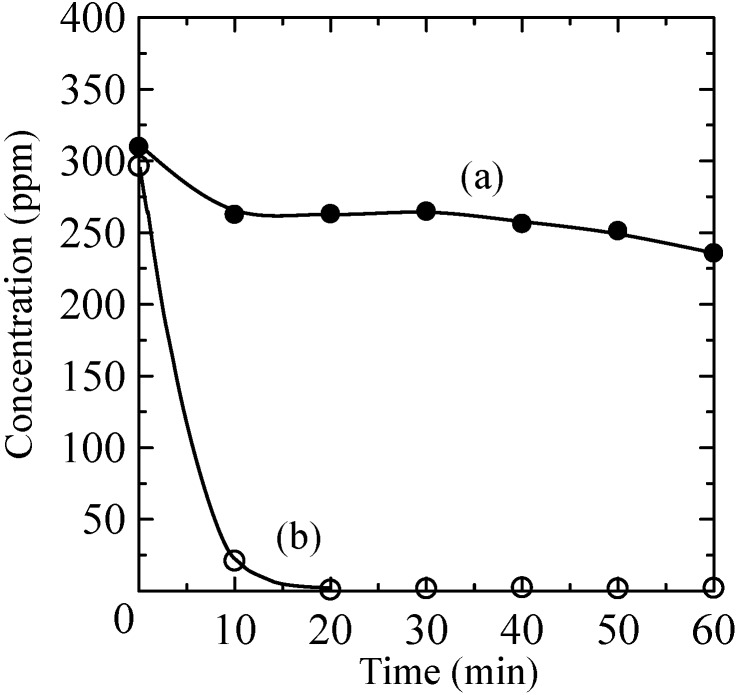
Change in the concentration of 2-propanol due to adsorption by the (a) TiO_2_-coated partially sintered glass and (b) TiO_2_-coated zeolite-porous glass composite.

Next, the adsorption of 2-propanol by the prepared composites was examined. The changes in the concentration of 2-propanol in the glass container with time under dark conditions are shown in Figure 6 for the TiO_2_-coated glass and the TiO_2_ coated zeolite-porous glass composite. The TiO_2_-coated glass adsorbed approximately 15% of the 2-propanol within the first 10 min, but then the concentration changed little up to 60 min. In contrast, the TiO_2_-coated zeolite-porous glass composite adsorbed most of the 2-propanol within 10 min because it had a much higher specific surface area than the TiO_2_-coated glass due to the presence of the X-zeolite.

### 2.4. Photocatalytic Properties of the Composites

The process of photocatalytic oxidation of 2-propanol has been well studied [54,61,62]. The reaction process is simply represented in Equations (1) and (2). Acetone is an intermediate product that is generated during the photocatalytic oxidative decomposition of 2-propanol:

C_3_H_7_OH + 1/2O_2_ → CH_3_COCH_3_ + H_2_O (1)

CH_3_COCH_3_ + 4O_2_ → 3CO_2_ + 3H_2_O (2)

**Figure 7:**
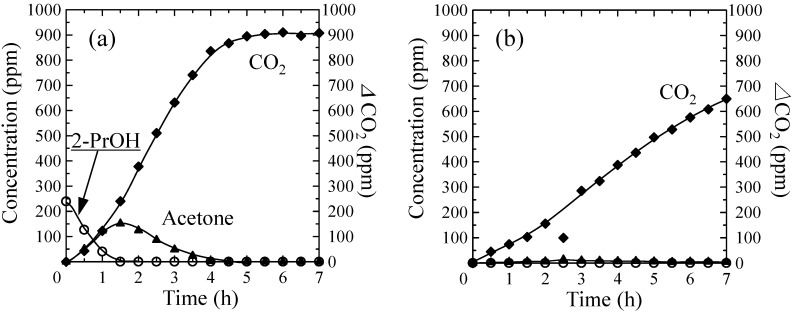
Changes in the concentrations of 2-propanol (open circles), acetone (filled triangles), and *Δ*CO_2_ (filled diamonds, right axis) for the (**a**) TiO_2_-coated partially sintered glass and (**b**) TiO_2_-coated zeolite-porous glass composite.

After adsorption of 2-propanol for 60 min, the samples were exposed to UV light irradiation, and the concentrations of 2-propanol, acetone, and CO_2_ were determined by gas chromatography. The changes in the concentrations of these gaseous species during UV light irradiation are shown in Figure 7a,b for the TiO_2_-coated partially sintered glass and the TiO_2_-coated zeolite-porous glass composite, respectively. The concentration of CO_2_ is presented as an increase over the initial concentration (*Δ*CO_2_) as indicated by the values of the coordinates on the right vertical axis. 

In Figure 7a, the concentration of the residual 2-propanol gradually decreased and fell below the detection limit for the partially sintered TiO_2_-coated glass after 1.5 h of UV light irradiation, while the concentration of acetone gradually increased to a maximum at 1.5 h, and then gradually decreased and fell below the detection limit after 4 h. The *Δ*CO_2_ value also gradually increased to approximately 900 ppm and became saturated after approximately 5 h. This saturated value of approximately 900 ppm corresponded to the theoretical *Δ*CO_2_ value expected for the complete conversion of 300 ppm of initial 2-propanol to H_2_O and CO_2_ via oxidation and decomposition as described in Equations (1) and (2). 

With the TiO_2_-coated partially sintered glass, a decrease in residual 2-propanol occurred simultaneously with an increase in the acetone and CO_2_ concentrations, indicating that the 2-propanol was photocatalytically oxidized to acetone, a portion of which was immediately oxidized to CO_2_ (Equations (1) and (2)), and a portion of which was released to the atmosphere. This behavior can be attributed to the lower adsorption capacity of the TiO_2_-coated partially sintered glass.

In contrast, no desorption of 2-propanol to the atmosphere was detected for the TiO_2_-coated zeolite-porous glass composite as shown in Figure 7b, and only a slight amount of acetone was observed. In addition, the *Δ*CO_2_ value increased linearly with the UV light irradiation time. However, the generation rate for CO_2_ was lower than that for the TiO_2_-coated partially sintered glass, and the *Δ*CO_2_ value was limited to approximately 650 ppm after 7 h. Considering the adsorption properties of X-zeolite as described in the introduction, the adsorption of 2-propanol and acetone on the X-zeolite in the present composite proceeded due to the electrostatic interactions between the Na^+^ or K^+^ ions on the surface of the X-zeolite and the 2-propanol and acetone. On the other hand, the adsorption of 2-propanol by the porous glass is considered to occur via hydrogen bonding, because Si-OH groups are likely to be present on the surface of the porous glass following hydrothermal treatment. However, based on the results of the FE-SEM analyses of the TiO_2_-coated samples (Figure 2 and Figure 4) and the decrease in the adsorption volume in the high relative pressure region (Figure 5), it is considered that the quantity of Si-OH groups decreased due to the subsequent repeated heat treatments at 400 °C and 500 °C necessary for fabrication of the TiO_2_ coating and thick TiO_2_ layer, respectively. Therefore, it can be concluded that the X-zeolite in the composite was the main adsorbent for the 2-propanol. 

**Figure 8:**
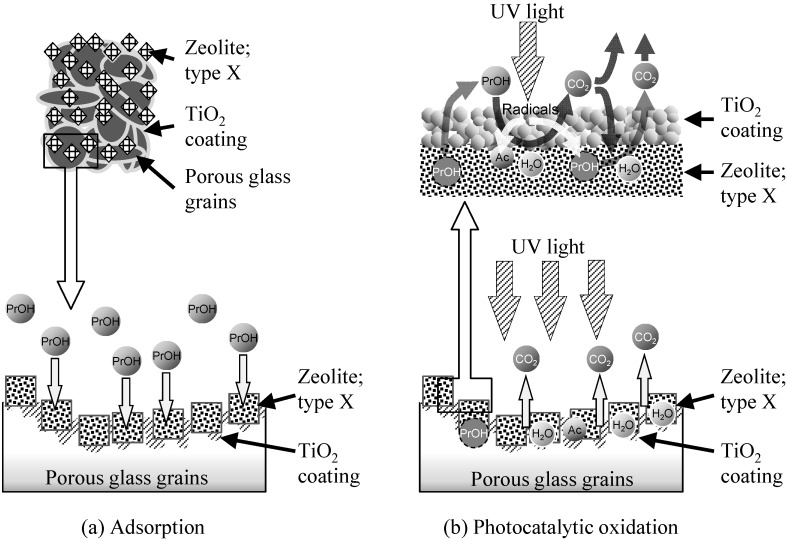
Schematic of (**a**) adsorption and (**b**) photocatalytic oxidation process by the TiO_2_-coated zeolite-porous glass composite.

The schematic of the structure of the TiO_2_-coated zeolite-porous glass composite are illustrated in Figure 8a,b in order to show its behaviors of adsorption and photocatalytic oxidation of 2-propanol and acetone, respectively. Even though CO_2_ is not a polar molecule, it has a quadrupole moment and can thus also be adsorbed on X-zeolites via electrostatic interactions [63]. The adsorption behavior of the X-zeolite for 2-propanol, acetone, and CO_2_ resulted in the slow generation rate of CO_2_ from the zeolite-porous glass composite. In other words, although the X-zeolite strongly adsorbed 2-propanol and acetone, a certain amount of the CO_2_ generated during irradiation with UV light was also adsorbed as shown in Figure 8b. These results suggest that the 2-propanol and acetone that desorbed from the X-zeolite (and the porous glass) were immediately oxidized by the TiO_2_, which was adjacent to the X-zeolite. Alternatively, the 2-propanol and acetone adsorbed in the X-zeolite may have been attacked by oxygen radicals generated on the TiO_2_ following irradiation with UV light. This oxidation process is possible to occur because the photocatalytic oxidation of 2-propanol sufficiently proceeded in the mechanically mixed zeolite and TiO_2_ powder system [55], and in our composite system, the diffusion distance was enough short for the radicals generated from TiO_2_ to reach the molecules adsorbed in the neighboring X-zeolite. Consequently, the TiO_2_-coated zeolite-porous glass composite could sufficiently remove 2-propanol by the adsorption and the photocatalytic oxidation and also it could suppress the release of acetone to the atmosphere during the photocatalytic decomposition process owing to its high adsorption capacity.

## 3. Experimental Section

### 3.1. Preparation of Partially Sintered Glass Supports

Porous silicate glass prepared using the phase separation phenomenon of oxide glasses was selected as the support. The sodium borosilicate glass system is considered to have a metastable immiscibility region and to separate into SiO_2_-rich and Na_2_O-B_2_O_3_ rich phases following appropriate thermal treatment above the glass transition temperature. In addition, SiO_2_-rich porous glass can be obtained from such phase separated glasses with spinodal decomposition compositions upon further thermal treatment and subsequent selective leaching of the Na_2_O-B_2_O_3_ rich phase [64]. Such a porous glass has been utilized as the support for TiO_2_ photocatalysts [17,18]. However, for the synthesis of zeolites, it is necessary to use a hydrothermal treatment process in highly concentrated alkaline solution. Because typical SiO_2_-rich glass has poor chemical resistance to alkaline solutions, it was necessary to use the mother glass, which has high durability in alkaline solutions, for synthesis of the glass-zeolite composite. Yazawa *et al.* reported that the Na_2_O-B_2_O_3_-CaO-ZrO_2_-Al_2_O_3_-SiO_2_ glass system has a metastable immiscibility region, and the porous glass obtained from this system exhibited high durability in alkaline solutions [57]. Based on these results, a glass batch composition for the mother glass of 5.7Na_2_O-9.2CaO-2.3Al_2_O_3_-3.2ZrO_2_-22.7B_2_O_3_-56.9SiO_2_ (mol %) was used in the present study.

Reagent-grade Na_2_CO_3_, H_3_BO_3_, SiO_2_, CaCO_3_, ZrO_2_, and Al_2_O_3_ (Wako Pure Chemical, Osaka, Japan) were used for glass preparation without further purification. The glass batch was prepared by mixing the weighed raw materials and melting the mixture in an alumina crucible at 1500 °C for 1 h in air. The melt was then quenched and immediately heat-treated at 750 °C for 12 h to allow phase separation to occur. Next, the partially sintered glass support was prepared by first grinding the annealed, heat-treated (phase separated) glass into 106–150 μm diameter particles and then forming the particles into a rod-like shape. The rod was sintered at 700 °C for 1 h, and then disk shapes were cut, each with a diameter of approximately 11 mm, a thickness of 1 mm, and a weight of 0.1 g.

### 3.2. Synthesis of the Zeolite on the Partially Sintered Glass Support

To form micro pores on the partially sintered glass disks, hydrothermal treatment was used to simultaneously etch the phase separated glass particles that formed the disk and synthesize the zeolite.

FAU type zeolites are well known to be synthesized by use of a mixed NaOH-KOH solution [65]. Precise preparation conditions of X-zeolite on the phase separated glass were determined based on the results of the preliminary experiments and were described as followings. Two partially sintered glass disks, one on top of the other, were placed in an aqueous solution containing NaOH (3 mol/L, 25 mL) and KOH (4 mol/L, 5 mL). Next, silica gel (0.4 g) and NaAlO_2_ (0.4 g), both purchased from Wako Pure Chemical and used without further purification, were added as the silica and alumina sources, respectively. The sample was tightly closed in a Teflon container (diameter: 35 mm; height: 55 cm in inner dimension; volume: approximately 50 mL) and was maintained at 75 °C for 24 h with stirring at 500 rpm. The hydrothermally treated sample was then washed with distilled water until the pH of the rinse water was approximately 8 and finally dried at 60 °C for 24 h.

### 3.3. TiO_2_ Coating of the Samples

TiO_2_ thin films were coated on the zeolite-glass composite and partially sintered glass using titanium diisopropoxide bis(acetylacetonate) [(CH_3_)_2_CHO]_2_Ti(C_5_H_7_O_2_)_2_; TPA, Aldrich, St. Louis, MO, USA) as the TiO_2_ source. A 2-propanol solution of TPA (1 mass% Ti) was stirred at approximately 2 °C for 1 h. Next, 200 μL of the TPA solution was dropped into the sample, dried at room temperature for 5 min, and subsequently heated at 400 °C for 10 min. This dropping and subsequent drying/heating cycle was repeated 6 times. Finally, the samples were heated at 500 °C for 2 h.

### 3.4. Characterizations of the Samples

The crystalline phases precipitated in the samples were examined using X-ray diffraction (XRD) analysis (LabX XRD-6100, Shimadzu, Kyoto, Japan) with a Cu-K_α_ radiation source. The micro textures of the surfaces and the cross sections of the samples were observed via field emission scanning electron microscopy (FE-SEM; S-4200, Hitachi, Tokyo, Japan) after platinum sputter coating. The adsorption and desorption isotherms and multipoint BET specific surface areas (Sg) of the samples were determined using the nitrogen adsorption technique (BELSORP-mini II, Nippon Bell, Osaka, Japan). The samples were heated at 200 °C for 24 h in a vacuum prior to measurement.

### 3.5. Gas Adsorption Ability and Photocatalytic Activity of the Samples

A calibrated gas generator (Permeater PD-1B, Gastech, Kanagawa, Japan) and dried air were used to produce air-diluted 2-propanol gas (approximately 300 ppm). A glass container (diameter: 4 cm; height: 6 cm; volume: approximately 65 mL) was placed in a glove bag filled with the air-diluted 2-propanol gas, and then the container filled with the same 2-propanol gas, immediately sealed and kept in the dark at 20 °C. The concentration of 2-propanol was determined using a gas chromatograph (GC, GC-8A, Shimadzu) with a thermal conductivity detector (TCD), a porous polymer beads column (Sunpak-A, 2 m, 160 °C, Shinwa Chemical, Kyoto, Japan), and a He carrier gas (20 mL/min). The gas in the sealed container was sampled (1 mL) every 10 min for 60 min using a micro syringe. After determination of the gas adsorption ability, the glass bottle was irradiation with UV light from a black light lamp (FL15BLB, peak wavelength = 0.30 mW/cm^2^ at 365 nm). The concentrations of 2-propanol, acetone, and CO_2_ in the glass container were determined every 30 min for 7 h using the same GC procedure.

## 4. Conclusions

A TiO_2_-zeolite-porous glass composite was prepared using melt-quenching for the glass preparation, hydrothermal treatment for the synthesis of the X-zeolite, and drop coating for deposition of the TiO_2_ thin film. The obtained composite comprised anatase phase TiO_2_, X-zeolite, and the porous glass, which were combined at the micro to nanometer scale. Synthesis of the X-zeolite on the porous glass support was possible because a glass with high resistance to alkaline solutions was used. In addition, the TiO_2_ and X-zeolite were solidified and formed into a disk shape with a relatively high specific surface area, and importantly, the TiO_2_ as the photocatalyst was adjacent to the X-zeolite as the adsorbent. Furthermore, the X-zeolite in the composite was very effective for the adsorption of polar molecules, such as 2-propanol as a model pollutant and acetone and its oxidized intermediate. The suppression of the release of acetone due to the presence of the X-zeolite suggested that the combined use of TiO_2_ and an adequate adsorbent is very useful for decreasing the emission of harmful intermediates generated during photocatalytic oxidative decomposition. Finally, the phase separated glass used as the support for the loading of the zeolite and TiO_2_ made it possible to prepare a bulk photocatalyst-adsorbent composite with superior adsorption and photo degradation properties for 2-propanol, and it was considered to be a good candidate for a composite of photocatalyst and adsorbent for the practical applications of the removal of gaseous organic pollutants.
